# Tacrolimus as Single-Agent Immunotherapy and Minimal Manifestation Status in Nonthymoma Myasthenia Gravis

**DOI:** 10.1155/2021/9138548

**Published:** 2021-11-20

**Authors:** Weiwei Duan, Yuyao Peng, Wanlin Jin, Song Ouyang, Huan Yang

**Affiliations:** ^1^Department of Neurology, Xiangya Hospital, Central South University, Changsha, 410008 Hunan, China; ^2^Department of Neurology, The First Hospital of Changsha, University of South China, Changsha, 410005 Hunan, China

## Abstract

**Background:**

Tacrolimus is a second-line immunosuppressant in myasthenia gravis (MG) therapy, which is mainly used in combination with corticosteroids to reduce steroid dose and maintain the effect of immunotherapy. However, few studies have focused on the effect of tacrolimus as single-agent immunotherapy on achieving minimal manifestation status (MMS). Thus, this study is aimed at exploring the efficacy and influencing factors of tacrolimus as single-agent immunotherapy in MG.

**Methods:**

Clinical data of 75 nonthymoma MG patients treated with tacrolimus single-agent as initial immunotherapy were retrospectively analyzed. The therapeutic effect was evaluated by Myasthenia Gravis Foundation of America postintervention status. Clinical factors affecting the achievement of MMS and treatment reactivity of different MG subtypes were determined by Cox regression analysis.

**Results:**

Tacrolimus was generally safe, with only two patients (2.7%) switching medications due to side effects. 32% of patients had improved symptoms after 1 month of treatment. 69.2% of patients achieved MMS or better after one year. The age < 39 years old, QMG score < 11 points, and AChR − Ab titer < 8.07 nmol/L were indicative of a favorable response, which was independent of gender, course of the disease. As for MG subtypes, ocular and seronegative MG showed better treatment sensitivity.

**Conclusions:**

Tacrolimus as single-agent immunotherapy takes effect quickly and can effectively enable nonthymoma MG patients to achieve MMS. Tacrolimus can be used alone for the initial immunotherapy of MG patients, especially for young, mild, and low antibody titer patients.

## 1. Introduction

Myasthenia gravis (MG) is an autoimmune disease that is mediated by autoantibodies and involves neuromuscular junctions [1]. It has mostly female predominance [2], ocular muscle weakness is the most common first symptom, and gradually extends to limb, bulbar, and ventilatory muscles, resulting in generalized MG (GMG) [3]. A series of autoantibodies (Abs) related to MG have been found, such as acetylcholine receptor (AChR), muscle-specific kinase (MuSK), and low-density lipoprotein receptor-related protein 4 (LRP4) Abs [4]. The exact pathogenesis of MG is still unclear. It is currently believed that the thymus plays an important role, and thymoma can be found in a considerable number of patients [5].

As an autoimmune disease, MG patients need long-term immunosuppressive treatment. Corticosteroids have been used for the treatment of MG since the 1960s and remain in widespread use today as first-line therapy [6]. However, a portion of MG patients may experience worsening symptoms or even a myasthenic crisis within the first 2 to 3 weeks after initiation of corticosteroid therapy. In addition, their long-term use is complicated by severe and often intolerable adverse effects including weight gain, dyslipidemia, cushingoid features, glaucoma, cataracts, osteoporosis, diabetes mellitus, avascular necrosis of the femoral head, and hypertension [7]. Due to concerns with adverse effects of chronic corticosteroid therapy, patients often need to take medications such as calcium, vitamin D, and gastric protectants [8]. In addition, corticosteroids also need to be gradually reduced and usually combine with steroid-sparing immunosuppressive drugs to prevent disease flares during dose tapering. This greatly increases the burden of medication for patients and may bring new side effects. Moreover, patients with metabolic syndromes such as hypertension and diabetes, and those who refuse to use corticosteroids due to concerns about their side effects, also pose limitations and challenges to their clinical use. Therefore, it is of great clinical value to explore new immunotherapy regimens for MG.

Tacrolimus is a macrolide lactone isolated from Streptomyces tsukubaensis, which blocks T cell activation by specifically inhibiting calcineurin [9, 10]. It was initially used for organ transplantation and then gradually adopted for the immunomodulatory treatment of autoimmune diseases. As a nonsteroidal immunosuppressant, it has also been widely used in the treatment of MG [6]. At present, it is mainly used as a steroid-sparing agent, but few studies have focused on the efficacy of tacrolimus as single-agent immunotherapy on achieving the therapeutic target (minimal manifestation status or better) [11] in MG, and no studies have investigated the differences in MG subtypes in response to treatment and the influence of patients' baseline clinical characteristics on treatment sensitivity. Thus, this study conducted a retrospective analysis to evaluate whether tacrolimus as single-agent immunotherapy contributes to the achievement of minimal manifestation status (MMS) in MG patients.

## 2. Methods

### 2.1. Data Source

We collected clinical data from 90 MG patients treated with tacrolimus single-agent as initial immunotherapy in the Department of Neurology, Xiangya Hospital, Central South University from July 2017 to June 2020. In order to eliminate the effect of thymectomy on the efficacy assessment, patients with thymoma and those who had undergone thymectomy within 48 months were excluded from the study. Finally, 75 patients were included in the study. These patients had not received other immunotherapy including steroids prior to tacrolimus intervention. The diagnosis of MG was made based on the following criteria: (a) fluctuating and fatigable muscle weakness; (b) at least two positive results of the following tests: neostigmine test, serum antibody assay, and repetitive nerve stimulation (RNS) test. AChR-Ab titer was detected using AChR-Ab ELISA Kit (RSR Ltd., Cardiff, UK), with a concentration of ≥0.45 nmol/L defined as positive.

Medical records of all patients were reviewed to collect clinical data before treatment including age, gender, course of disease, serum autoantibodies status, Myasthenia Gravis Foundation of America (MGFA) classification (MGFA I or II stands for mild weakness status and III or IV represents more severe weakness) [12], and the quantitative myasthenia gravis score (QMGs) [13], as well as follow-up data including MGFA postintervention status (MFGA PIS) [12] and side effects. The ethics committee of Xiangya Hospital approved the study protocol.

### 2.2. Tacrolimus Administration Regimen

The starting dose of tacrolimus was 1 mg each time, twice a day. A satisfactory serum concentration (4.8-10 ng/ml) [14] was obtained by adjusting the dose or adding Wuzhi capsule [15]. In addition, the dose adjustment took into account the efficacy and patient tolerance. The maintenance dose of tacrolimus was 1-3 mg per day. No other immunosuppressive drugs including steroids were used. The use of cholinesterase inhibitors such as pyridostigmine bromide was permitted.

### 2.3. Efficacy and Safety Evaluation

The efficacy was evaluated by the MGFA PIS. MGFA PIS was designed to assess the clinical state of MG patients at any time after the institution of treatment for MG, which includes complete stable remission (CSR), pharmacologic remission (PR), MMS, improved, unchanged, worse, exacerbation, and died of MG [12]. The follow-up time points we selected were 1 month, 3 months, 6 months, and 12 months.

To evaluate the safety of tacrolimus, we collected posttreatment data, including symptoms and signs, as well as the results of laboratory tests such as complete blood count, liver and kidney function, blood glucose, and electrolytes that were regularly monitored.

### 2.4. Statistical Analysis

All statistical analysis was performed with SPSS v26.0. Categorical variables were expressed as counts and percentages. Continuous data were expressed as mean ± standard deviation (SD). Kaplan-Meier (K-M) analysis was used to estimate the probability of achieving MMS or better. Cox regression analysis was adopted to identify the predictors of efficacy and assess the response of different MG subtypes to treatment. Cox regression analysis and Cox model variables were tested using the proportional hazards (PH) assumption. The log minus log survival curve plot method was used for categorical variables, and the two curves do not cross, indicating that the variable satisfies the PH assumption. For continuous variables, the Schoenfeld residual method was adopted. If the *p* value is less than 0.05, the variable meets the assumption. Chi-square test or Fisher's exact test was used to compare qualitative data. X-tile, a bioinformatics tool for outcome-based cut-point optimization, was used to determine the cut-off value [16]. In this study, *p* value < 0.05 was considered significant.

## 3. Results

### 3.1. Patient Characteristics

**Figure 1:**
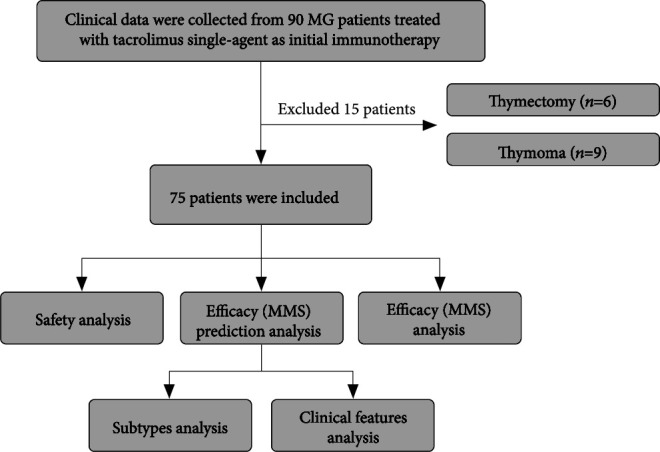
The flowchart designed for the study.

The workflow designed for the study was shown in Figure 1. A total of 75 nonthymoma MG patients who chose tacrolimus single-agent as initial immunotherapy were enrolled in this study. The mean age was 41.61 ± 17.95 years old. Male accounted for 38.7% (*n* = 29) and female accounted for 61.3% (*n* = 46). The average course of disease (time from onset to initiation of tacrolimus treatment) was 50.14 ± 85.57 months. And the mean QMG score before treatment was 10.17 ± 4.73 points. The MGFA classification was as follows: MGFA I (*n* = 27, 36.0%), MGFA II (*n* = 35, 46.7%), MGFA III (*n* = 10, 13.3%), and MGFA IV (*n* = 3, 4.0%). In terms of MG-related autoantibodies, AChR-Ab was positive in 58 cases (77.3%) and negative in 17 cases (22.7%); 2 cases (2.7%) were positive for MuSK-Ab and 73 cases (97.3%) were negative; no anti-LRP4-Ab positive patients were found. 16 (21.3%) patients were accompanied by autoimmune thyroid disorders (ATD) (6 cases with hyperthyroidism, 3 cases with hypothyroidism, and 7 cases with antithyroid antibodies positive only). The baseline characteristics of patients were summarized in Table 1.

### 3.2. Safety of Tacrolimus

In general, tacrolimus was safe, and no serious adverse reactions were observed. In 75 patients, 8 (10.7%) patients experienced adverse reactions. The side effects of most patients could be alleviated through dose adjustment and symptomatic treatment. 2 patients (2.7%) switched to other treatment regimens due to adverse reactions. The most common side effects were metabolic disorders and gastrointestinal reactions, including hyperuricemia, elevated blood glucose, and nausea. The tacrolimus-related side effects were summarized in Table 2.

### 3.3. Efficacy of Tacrolimus as Single-Agent Immunotherapy on Achieving MMS

The follow-up time was 12 months. Due to some patients were lost to follow-up, the number of patients at each follow-up time point was 75 (1 month), 62 (3 months), 49 (6 months), and 39 (12 months). We compared the proportion of patients with different MGFA PIS at each follow-up time point. At 1 month, the percentage of patients with unchanged was the highest (62.7%), and the difference was statistically significant compared with other MGFA PIS (*p* < 0.05). At 3 months, the percentage of patients with improved was the highest (40.3%), but the difference with other MGFA PIS was not statistically significant (*p* > 0.05). At 6 months, the highest proportion (44.9%, *p* > 0.05) was present in patients with MMS or better. At 12 months, patients with MMS or better also accounted for the highest proportion (69.2%, *p* < 0.05) (Table 3).

With MMS or better as treatment outcome, the proportion of patients achieving treatment endpoints at each time point was 6.7% (5/75, MMS 6.7%) after 1 month, 19.3% (12/62, MMS 17.7%, PR 1.6%) after 3 months, 44.9% (22/49, MMS 38.8%, PR 6.1%) after 6 months, and 69.2% (27/39, MMS 48.7%, PR 20.5%) after 12 months. In addition, the proportion of achieving improved at each time point was 25.3% (19/75, 1 month), 40.3% (25/62, 3 months), 28.6% (14/49, 6 months), and 12.8% (5/39, 12 months). 8 (10.4%) patients changed their treatment plan due to poor treatment effect, or added corticosteroid, or changed to mycophenolate mofetil (MMF), or received intravenous immunoglobulin and plasma exchange. No patient died during the treatment. The proportion of patients reaching each MGFA PIS at each follow-up time point was shown in Table 3.

In addition, we predicted the probability of MG patients achieving MMS or better at each time point using K-M analysis. The results demonstrated that the cumulative probability of achieving MMS or better was 6.7% (95% CI 1.0-12.4%) at 1 month, 18.1% (95% CI 8.7-27.5%) at 3 months, 40.3% (95% CI 26.8-53.8%) at 6 months, and 57.8% (95% CI 41.7-73.9%) at 12 months (Supplementary materials: Figure S1).

### 3.4. Clinical Features Correlated with Efficacy

In order to understand the impact of patients' baseline clinical characteristics on achieving MMS, we performed a Cox regression analysis with MMS or better as the outcome index, including age, gender, course of disease, AChR-Ab titer, baseline QMG score, and MGFA classification. PH assumption testing showed that these variables satisfied the assumption and could be incorporated into the model (Supplementary materials: Figure S2 and Table S1). The follow-up time was focusing on 12 months when the Cox regression model was applied. Censored cases were defined as those that did not reach MMS or better during the follow-up period, which accounted for 64% (48/75) of all cases. The results showed that age, QMG score, and AChR-Ab titer were factors that affected patients to achieve MMS, regardless of gender and course of disease (*p* > 0.05, Table 4). It was further determined that the cut-off value of age was 39 years old, QMG score was 11 points, and Ab titer was 8.07 nmol/L by X-tile software (Supplementary materials: Figure S3). Age ≥ 39 (*p* = 0.014, HR 0.379), QMG score ≥ 11 points (*p* = 0.025, HR 0.353), and AChR − Ab titer ≥ 8.07 nmol/L (*p* = 0.009, HR 0.298) suggested a poor response to treatment (Table 4). And MGFA I was a benign factor related to MMS (*p* = 0.018, HR 2.505, Table 4).

### 3.5. Differential Sensitivity of MG Subtypes to Tacrolimus

**Figure 2:**
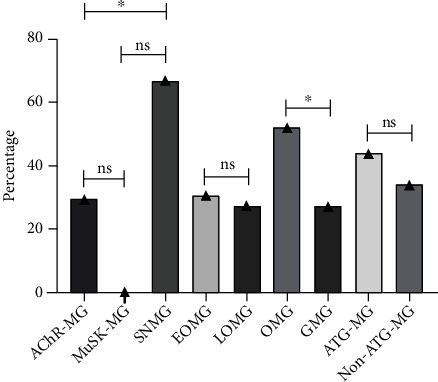
The proportion of each MG subtype reaching minimal manifestations status or better. Chi-square test was used for comparisons between AChR-MG and SNMG, EOMG and LOMG, OMG and GMG, and ATG-MG and non-ATG-MG. The comparisons between AChR-MG and MuSK-MG, SNMG, and MuSK-MG were performed using Fisher's exact test. ∗*p* < 0.05; ns: no significance.

We analyzed the reactivity of different MG subtypes to tacrolimus. The 75 patients were divided into 9 subgroups: AChR antibody-positive MG (AChR-MG, *n* = 58), early-onset (EOMG, *n* = 36), late-onset (LOMG, *n* = 22), MuSK antibody-positive MG (MuSK-MG, *n* = 2), seronegative (SNMG, *n* = 15), ocular MG (OMG, *n* = 27), and generalized MG (GMG, *n* = 48), MG with autoimmune thyroid disorders (ATD-MG, *n* = 16), and MG without autoimmune thyroid disorders (non-ATD-MG, *n* = 59) [4]. The proportion of patients reaching MMS or better in each subgroup was 29.3% (17/58, AChR-MG), 30.6% (11/36, EOMG), 27.3% (6/22, LOMG), 0% (0/2, MuSK-MG), 66.7% (10/15, SNMG), 51.9% (14/27, OMG), 27.1% (13/48, GMG), 43.8% (7/16, ATD-MG), and 33.9% (20/59, non-ATD-MG), respectively. As shown in Figure 2, compared with AChR-MG subtypes, the proportion of SNMG patients achieving MMS or better was higher (*p* = 0.014), and there was no significant difference between AChR-MG and MuSK-MG, and SNMG and MuSK-MG (*p* > 0.05). And the proportion of OMG patients with MMS or better was higher than that of GMG patients (*p* = 0.03). No statistical difference was found between EOMG and LOMG, and ATD-MG and non-ATD-MG (*p* > 0.05). We further evaluated the effect of MG subtypes on MMS attainment by Cox regression analysis. We found that SNMG (*p* = 0.006, HR 3.013) and OMG (*p* = 0.018, HR 2.505) were benign factors in the implementation of MMS, while AChR-MG (*p* = 0.031, HR 0.422) and GMG (*p* = 0.018, HR 0.399) indicated relatively poor treatment response (Table 5). No correlation was found with MuSK-MG, LOMG, EOMG, ATD-MG, and non-ATD-MG (*p* > 0.05, Table 5). The above results indicate that OMG and SNMG subtypes respond better to tacrolimus as single-agent immunotherapy.

## 4. Discussion

Tacrolimus, as a nonsteroidal immunosuppressant, has a beneficial effect in the treatment of MG [17]. Tacrolimus is usually used in combination with corticosteroid and acts as a steroid-sparing to reduce the corticosteroid burden in patients. However, patients still need to use corticosteroids for a long time, which inevitably leads to some side effects of steroids [18]. The application of tacrolimus as single-agent immunotherapy in other immune-related diseases has been studied. In adult-onset minimal change nephrotic syndrome, tacrolimus as single-agent immunotherapy was found to be noninferior to conventional glucocorticoid treatment [19]. In another multicenter, randomized, controlled trial for adults with de novo minimal change disease, there were no significant differences between the tacrolimus and prednisolone treatment cohorts in the proportion of patients in complete remission at 8, 16, and 26 weeks. The difference in relapse rates for patients between two groups who achieved complete remission and in the time from complete remission to relapse also was not found [20]. In addition, it may be a therapeutic option in patients with moderate-to-severe active refractory ulcerative colitis [21] and appears to be safe and efficacious in heart transplant recipients [22]. In patients with rheumatoid arthritis, when conventional disease-modifying antirheumatic drugs have failed or biological agents are not an option, tacrolimus could be an alternative option [23]. However, few studies have focused on the effect of tacrolimus as single-agent immunotherapy on achieving minimal manifestations status in MG.

In this study, we retrospectively analyzed the clinical data of nonthymoma MG patients who received tacrolimus single-agent as initial immunotherapy. We found that 32% of patients had improved symptoms after 1 month of treatment, which indicates that tacrolimus as single-agent immunotherapy used for the treatment of MG has a rapid onset. This is its advantage compared to other steroid-sparing immunosuppressive drugs, such as azathioprine (AZA), the current first-line immunosuppressant for the treatment of MG, usually takes effect after a few months [24]. And MMF, another immunosuppressant commonly used in MG treatment, generally begins to improve MG after 6 months, both with prednisone and used alone [25]. As treatment progressed, the proportion of patients who reached MMS increased. 69.2% of patients reached MMs or better after one year. Only 2.7% of patients changed their medications due to side effects. These results suggest that tacrolimus is effective and safe as single-agent immunotherapy for MG and can contribute to the realization of MMS in patients. The clinical factors affecting the achievement of MMS in tacrolimus treatment were further explored. We found that patients with age < 39, QMG score < 11 points, and AChR − Ab titer < 8.07 nmol/L had a better response to treatment. These results suggest that patients with young, mild, and low antibody titer are more likely to benefit from tacrolimus as single-agent immunotherapy.

This study also found that the efficacy of tacrolimus was antibody selective, and negative serum antibodies indicate better treatment response than AChR antibody positive. This may be related to the good treatment sensitivity of SNMG patients [26]. This also suggests tacrolimus as single-agent immunotherapy has some limitations in the clinical application, since AChR-MG accounts for the majority of patients. However, we believe AChR antibody-positive should not be a barrier to selecting tacrolimus. In this study, approximately a quarter of AChR-MG patients still achieved satisfactory treatment outcomes (MMS or better), and the limited sample size and loss of follow-up patients also affected the observation of treatment prognosis. Therefore, for AChR-MG patients, whether to initiate tacrolimus should take into consideration comprehensively the patient's condition as well as the responsiveness and tolerance to other immunosuppressants. This study suggests that tacrolimus as single-agent immunotherapy is more suitable for nonsevere AChR-MG patients. MuSK-MG is generally considered to be treatment-resistant [27], but no effect of MuSK antibody on treatment sensitivity was found in this study, which we believe is related to the small number of cases.

In MG, patients often present with ocular muscle involvement as the initial symptom (OMG) and gradually involve limb muscles, bulbar muscles, and respiratory muscles (GMG) as the disease progresses. In this study, the treatment response of OMG patients was better than that of GMG patients, suggesting that the timing of tacrolimus intervention is more suitable in the early stage of onset. Given that 27.1% of GMG patients achieved MMS or better during treatment, tacrolimus as single-agent immunotherapy can also be used as a treatment option for GMG patients.

In addition, other studies have found that patient response to tacrolimus is influenced by pharmacogenetics. MG patients with rs2069762 G/T and G/G genotype and TAGG haplotype for interleukin-2 gene tend to have a poor response to tacrolimus [28]. And the multiple single nucleotide polymorphisms on CYP3A4, CYP3A5, FKBP1A, and NFATC2 genes involved in the pharmacokinetics and pharmacodynamics of tacrolimus are closely related to therapeutic effect [29]. This indicates that the sensitivity of MG patients to tacrolimus treatment is affected by multiple factors.

In general, compared with a combination of tacrolimus and steroids, tacrolimus used alone avoids the aggravation that may be caused by the initial use of steroids and the side effects caused by long-term use and reduces the burden of patient's medication, and its effect is rapid and definite. Of course, there are some limitations in our study. First of all, this study was retrospective, without a control group, and the selection or misclassification bias could not be ruled out. Second, some patients were lost during the follow-up process, which inevitably affected the results. In addition, as a single-center study, the sample size was limited and MG patients with anti-LRP4 antibody were not included in this study. Thus, the results of this study need to be verified by a larger prospective control study.

## 5. Conclusions

Tacrolimus as single-agent immunotherapy is available for achieving minimal manifestations status in the treatment of MG. It can be used as an initial immunotherapy scheme, especially for young, mild, and low antibody titer patients.

## Figures and Tables

**Table 1:** Baseline characteristics of patients.

Characteristics	
Age (years, mean ± SD)	41.61 ± 17.95
Male/female (*n*, %)	29 (38.7%)/46 (61.3%)
Course of disease (months, mean ± SD)	50.14 ± 85.57
AChR-Ab (*n*, %)	
Positive	58 (77.3%)
Negative	17 (22.7%)
MuSK-Ab (*n*, %)	
Positive	2 (2.7%)
Negative	73 (97.3%)
LRP4-Ab (*n*, %)	
Positive	0 (0%)
Negative	75 (100%)
QMG score (mean ± SD)	10.17 ± 4.73
MGFA classification (*n*, %)	
I	27 (36.0%)
II	35 (46.7%)
III	10 (13.3%)
IV	3 (4.0%)
Autoimmune thyroid disorders (*n*, %)	
Yes	16 (21.3%)
No	59 (78.7%)

**Table 2:** Adverse events during tacrolimus treatment.

Adverse events	*n* (%)
Hyperuricemia	4 (5.3%)
Elevated blood glucose	2 (2.7%)
Nausea	2 (2.7%)
Hyperlipidemia	1 (1.3%)
Muscle cramps	1 (1.3%)
Palpitations	1 (1.3%)
Hand tremor	1 (1.3%)

**Table 3:** The number and proportion of patients reaching each MGFA PIS at each follow-up time point.

MGFA PIS	1 month (*n* = 75)	3 months (*n* = 62)	6 months (*n* = 49)	12 months (*n* = 39)
Worse	4 (5.3%)	4 (6.5%)	1 (2.0%)	0
Unchanged	47 (62.7%) ∗	21 (33.9%)	12 (24.5%)	7 (17.9%)
Improved	19 (25.3%)	25 (40.3%) ^ns^	14 (28.6%)	5 (12.8%)
MMS	5 (6.7%)	11 (17.7%)	${\left.\begin{array}{c} 19\;\left(38.8\%\right)\\ 3\;\left(6.1\%\right) \end{array}\right|}^{\text{ns}}$	${\left.\begin{array}{c} 19\;\left(48.7\%\right)\\ 8\;\left(20.5\%\right) \end{array}\right|}^{*}$
PR	0	1 (1.6%)		

Note: the proportion of different MGFA PIS at each follow-up time point was compared by nonparametric Chi-square test. ∗*p* < 0.05; ns: no significance.

**Table 4:** Cox regression analysis of factors affecting the response to tacrolimus.

	*p* value	HR	95% CI
Age ≥ 39 years	0.014∗	0.379	0.175-0.821
Gender (male)	0.600	0.792	0.332-1.890
Course of disease	0.485	0.998	0.994-1.003
AChR − Ab titer ≥ 8.07 nmol/L	0.009∗∗	0.298	0.120-0.740
QMG score ≥ 11 points	0.025∗	0.353	0.142-0.876
MGFA I	0.018∗	2.505	1.174-5.347
MGFA II	0.240	0.631	0.293-1.361
MGFA III	0.337	0.494	0.117-2.087
MGFA IV	0.498	0.047	0.00-326.48

Note: HR: hazard ratio; 95% CI: 95% confidence interval. ∗*p* < 0.05, ∗∗*p* < 0.01.

**Table 5:** Cox regression analysis of MG subtype treatment response.

Subtypes	*p* value	HR	95% CI
AChR-MG	0.031∗	0.422	0.193-0.923
MuSK-MG	0.512	0.047	0-442.901
SNMG	0.006∗∗	3.013	1.375-6.604
EOMG	0.820	0.915	0.424-1.974
LOMG	0.114	0.480	0.193-1.194
OMG	0.018∗	2.505	1.174-5.347
GMG	0.018∗	0.399	0.187-0.852
ATD-MG	0.929	0.961	0.406-2.277
Non-ATD-MG	0.929	1.04	0.439-2.463

Note: ∗*p* < 0.05, ∗∗*p* < 0.01.

## Data Availability

The data used and analyzed during this study are available from the corresponding author on reasonable request.

## Supplementary Materials

Supplementary Materials Figure S1: cumulative probability of achieving MMS or better in MG patients using tacrolimus as single-agent immunotherapy. Figure S2: the proportional hazard assumption for categorical variables was tested by plotting the log minus log survival curves. Figure S3: the optimal cut-off value of variable was determined by X-tile software. Table S1: correlation analysis between Schoenfeld residuals and time rank for continuous variables.
