# Supplementary material for: Tacrolimus as Single-Agent Immunotherapy and Minimal Manifestation Status in Nonthymoma Myasthenia Gravis
Source: J Immunol Res. 2021 Nov 20;2021:9138548. doi: 10.1155/2021/9138548 (PMC8627335; doi:10.1155/2021/9138548)
Supplement: Supplementary Materials — Figure S1: cumulative probability of achieving MMS or better in MG patients using tacrolimus as single-agent immunotherapy. Figure S2: the proportional hazard assumption for categorical variables was tested by plotting the log minus log survival curves. Figure S3: the optimal cut-off value of variable was determined by X-tile software. Table S1: correlation analysis between Schoenfeld residuals and time rank for continuous variables. [file 9138548.f1.docx]

Supplementary Materials

# Supplementary Figures





**Figure S1.** Cumulative probability of achieving MMS or better in MG patients using tacrolimus as single-agent immunotherapy. The cumulative probability was 6.7% (95%CI 1.0-12.4%) at 1 month, 18.1% (95%CI 8.7-27.5%) at 3 months, 40.3% (95%CI 26.8-53.8%) at 6 months, and 57.8% (95%CI 41.7-73.9%) at 12 months.

**

**

**Figure S2.** The proportional hazards assumption for categorical variables was tested by plotting the log minus log survival curves. The two curves do not intersect suggests that the assumption is true. **(A)** gender. **(B)** MGFA I. **(C)** MGFA II. **(D)** MGFA III. **(E)** MGFA IV.



**Figure S3.** The optimal cut-off value of variable was determined by X-tile software. (**A**) age. (**B**) AChR-Ab titer. (**C**) QMG score.

# Supplementary Table

**Table S1.** Correlation analysis between Schoenfeld residuals and time rank for continuous variables.

|  | **Pearson Correlation** | **p-value** |
| --- | --- | --- |
| **Course of disease** | -0.086 | 0.668 |
| **AChR-Ab titer** | 0.295 | 0.136 |
| **QMG score** | 0.312 | 0.113 |
| **Age** | 0.079 | 0.695 |

Note: p > 0.05 indicates the variable meets the PH assumption.
